# Germline whole exome sequencing of a family with appendiceal mucinous tumours presenting with pseudomyxoma peritonei

**DOI:** 10.1186/s12885-020-6705-y

**Published:** 2020-05-01

**Authors:** Mei Sim Lung, Catherine A. Mitchell, Maria A. Doyle, Andrew C. Lynch, Kylie L. Gorringe, David D. L. Bowtell, Australian Ovarian Cancer Study Group, Ian G. Campbell, Alison H. Trainer

**Affiliations:** 1 Research Division, Cancer Genetics Laboratory, Peter MacCallum Cancer Centre, Victorian Comprehensive Cancer Centre Building, 305 Grattan St., Melbourne, Victoria VIC 3000 Australia; 2 Department of Pathology, Peter MacCallum Cancer Centre, Melbourne, Victoria Australia; 3 Research Computing Facility, Peter MacCallum Cancer Centre, Melbourne, Victoria Australia; 4 Department of Surgical Oncology, Peter MacCallum Cancer Centre, Melbourne, Victoria Australia; 5 Cancer Genomics Program, Peter MacCallum Cancer Centre, Melbourne, Victoria Australia; 6 Cancer Genetics and Genomics Laboratory, Peter MacCallum Cancer Centre, Melbourne, Victoria Australia; 7 Sir Peter MacCallum Department of Oncology, University of Melbourne, Parkville, Victoria Australia; 8 Department of Biochemistry and Molecular Biology, University of Melbourne, Parkville, Victoria Australia; 9 Parkville Familial Cancer Centre, Peter MacCallum Cancer Centre, Melbourne, Victoria Australia; 10 Department of Medicine, University of Melbourne, Parkville, Victoria Australia

**Keywords:** Pseudomyxoma peritonei, Appendiceal tumour, Familial, Germline predisposition, Exome sequencing

## Abstract

**Background:**

Familial cases of appendiceal mucinous tumours (AMTs) are extremely rare and the underlying genetic aetiology uncertain. We identified potential predisposing germline genetic variants in a father and daughter with AMTs presenting with pseudomyxoma peritonei (PMP) and correlated these with regions of loss of heterozygosity (LOH) in the tumours.

**Methods:**

Through germline whole exome sequencing, we identified novel heterozygous loss-of-function (LoF) (i.e. nonsense, frameshift and essential splice site mutations) and missense variants shared between father and daughter, and validated all LoF variants, and missense variants with a Combined Annotation Dependent Depletion (CADD) scaled score of ≥10. Genome-wide copy number analysis was performed on tumour tissue from both individuals to identify regions of LOH.

**Results:**

Fifteen novel variants in 15 genes were shared by the father and daughter, including a nonsense mutation in *REEP5*. None of these germline variants were located in tumour regions of LOH shared by the father and daughter. Four genes (*EXOG*, *RANBP2, RANBP6 and TNFRSF1B*) harboured missense variants that fell in a region of LOH in the tumour from the father only, but none showed somatic loss of the wild type allele in the tumour. The *REEP5* gene was sequenced in 23 individuals with presumed sporadic AMTs or PMP; no LoF or rare missense germline variants were identified.

**Conclusion:**

Germline exome sequencing of a father and daughter with AMTs identified novel candidate predisposing genes. Further studies are required to clarify the role of these genes in familial AMTs.

## Background

Appendiceal mucinous tumours (AMTs) are rare, occurring at an age-adjusted incidence of 0.12 per million individuals, with a median age at diagnosis of 59 years and no gender bias [1]. Pseudomyxoma peritonei (PMP) is a clinical term describing gelatinous ascites, associated with the presence of mucin-producing cells within the peritoneal cavity, usually associated with an appendiceal mucinous neoplasm, either a low-grade appendiceal mucinous neoplasm (LAMN) or a mucinous

adenocarcinoma [2]. Whether PMP occurs in all, or just a molecular subset of AMTs is unknown.

Molecular studies of PMP and AMTs are limited, but somatic mutations have been identified in *KRAS (*60–100% of sequenced cases), *GNAS* (30–74%), *SMAD4* (16%), and *TP53* (5–14%) [3–9]. Compared to colorectal cancer, somatic mutations in the *APC* gene are observed at a much lower frequency, ranging between 0 and 33% [4, 7, 9, 10]. An *RNF43* stop-gained mutation in a region of LOH in a LAMN has recently been described [10]. In 5 LAMNs, a mutation signature consistent with deamination of 5-methylcytosine was commonly detected [10].

Familial forms of AMTs are rare, with only two reported cases in the literature. The first family comprised monozygotic twin brothers [11]. The first twin was diagnosed with PMP at the age of 35 during an umbilical hernia repair, and he was subsequently found to have a perforated AMT. After this diagnosis, his asymptomatic twin underwent a prophylactic appendectomy which identified a non-perforated AMT. Somatic LOH of the *APC* locus was identified in the second AMT but not in the original case. No germline *APC* mutation data were available.

The second family comprised a brother and sister diagnosed with AMTs at the ages of 69 and 77 years respectively [12]. The brother presented with acute appendicitis and an AMT was identified at surgery, while his sister presented with increasing abdominal girth and was found to have PMP and an AMT. An assessment for Lynch syndrome was performed in this family. The sister’s AMT had normal immunohistochemistry staining for the mismatch repair proteins MLH1, MSH2, MSH6 and PMS2, and her tumour was microsatellite stable, whilst her brother underwent constitutional mutation analysis of *MLH1, MSH2* and *MSH6* which did not identify a pathogenic variant.

**Fig. 1:**
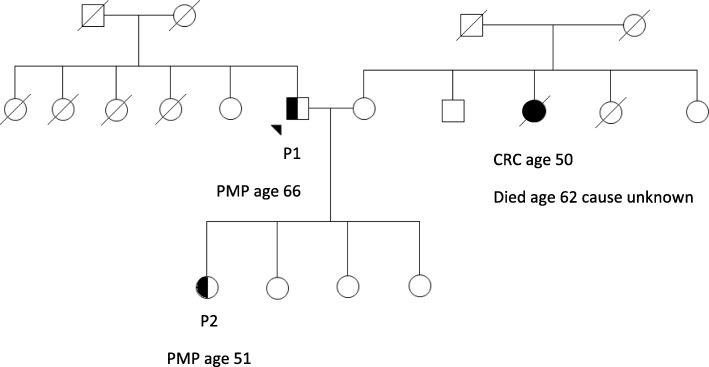
Pedigree of parent child pseudomyxoma peritonei (PMP). Legend. The father (P1) presented with PMP at age 66, while his daughter (P2) developed PMP at age 51

Here, we report the first familial parent-child PMP case, a father (P1) and daughter (P2) who were both diagnosed with PMP secondary to an AMT at the ages of 66 and 51 years respectively (Fig. 1). P1 was incidentally found to have PMP on staging CT for a Gleason 6 prostate cancer. A diagnosis of PMP was made on diagnostic laparotomy and he underwent drainage of a large abdominal cyst. Subsequent cytoreductive surgery undertaken 4 years later when he became symptomatic, identified a ruptured LAMN. His daughter (P2) presented with 6 months of menorrhagia, dysmenorhoea and pelvic and right upper quadrant discomfort. She was found to have PMP and a moderately differentiated mucinous adenocarcinoma of the appendix.

Identifying the germline genetic aetiology of rare familial colorectal cancer syndromes such as familial adenomatous polyposis has led to a better understanding of many somatic pathways and mechanisms underlying sporadic forms of the disease. Thus, we sought to identify predisposing genes in this family through a germline whole exome sequencing approach and looked for regions of LOH in their tumour tissues as a secondary filter for potentially pathogenic variants.

## Methods

### Ethics

All procedures performed involving human participants were in accordance with the ethical standards of the Peter MacCallum Cancer Centre Human Research and Ethics Committee (project number 10_83) and with the 1964 Helsinki declaration and its later amendments or comparable ethical standards. Written informed consent was obtained from all individual participants included in the study. P1 and P2 signed patient information and consent forms to participate in this study, which included the consent for publication of de-identified data.

### Whole exome sequencing

One μg of germline DNA was obtained from peripheral leucocytes and fragmented using the Covaris S2 System (Covaris, Woburn, MA, USA). The SureSelect Human All Exon v1 (Agilent, Santa Clara, CA, USA) was used for exome enrichment according to the manufacturer’s protocol. Paired-end 100 base pair reads were sequenced on a HiSeq2000 (Illumina Inc., San Diego, CA, USA) instrument. Both exomes passed sequencing quality control with mean target base coverages of 129x and 121x for P1 and P2 respectively and > 95% of targeted bases covered more than 10x.

### Sequence alignment and variant calling

Raw sequence reads were quality checked with FastQC [13] and trimmed for low quality bases and adaptor if necessary using Cutadapt [14]. Reads were aligned to the human genome (GRCh37 assembly) using BWA-MEM [15]. Duplicate reads were marked using Picard [16] followed by merging of BAM files for both individuals. Local realignment around indels was performed on the merged BAM files using the Genome Analysis Tool Kit (GATK) software v3.1 [17]. Subsequently, base quality score recalibration was performed using GATK software. Single nucleotide variants (SNVs) and indels were identified using the GATK HaplotypeCaller and annotated with information from Ensembl release 73 using Ensembl’s Perl API and Variant Effect Predictor [18, 19]. Each variant was annotated with its frequency in the 1000 Genomes Project [20], the National Heart, Lung and Blood Institute (NHLBI) Grand Opportunity (GO) Exome Sequencing Project [21] and an in-house exome dataset of 147 familial breast cancer cases [22]. The likely pathogenic consequence for each variant was determined by Polyphen [23], SIFT [24], and Combined Annotation Dependent Depletion (CADD) scaled score [25].

### Exome data analysis

For genes with multiple transcripts, transcripts were prioritised on 1) most to least deleterious predicted impact of variant on protein function (Supplementary Data 1 and 2) RefSeq transcript. The highest ranking transcript was taken forward for further analysis. LoF variants and missense variants which met the following criteria were considered for further analysis: [1] Phred variant quality score of > 30, and [2] variant allele frequency between 0.15 and 0.8. For identification of novel variants shared between P1 and P2, variants were excluded if they were present in control cohorts: 1000 Genomes Project, NHLBI GO Exome Sequencing Project or an in-house cohort of 147 Australian familial breast cancer exomes. All loss-of-function (LoF) variants (truncating frameshift, nonsense, essential splice site), and missense variants with a CADD scaled score ≥ 10 were manually checked in the Integrated Genome Viewer (IGV) [26, 27].

Variants shared between P1 and P2 which were confirmed on Sanger sequencing were checked in the Genome Aggregation Database v2 dataset (gnomAD) [28], comprising exome and genome data from 125,748 and 15,708 unrelated individuals respectively, for the population frequency, to confirm that these variants were rare or novel. Variants with a frequency greater than 1X 10^− 4^ in the gnomAD dataset were considered too common to account for the development of AMTs and were excluded.

### Whole genome amplification and Sanger  sequencing

Candidate variants were confirmed by Sanger sequencing using whole-genome amplified DNA from P2. Whole-genome amplification of genomic DNA was performed using the REPLI-g Midi Kit (Qiagen, Redwood City, CA, USA). PCR primers were designed using the Primer3 program v0.4.0 [29, 30] and are listed in Supplementary Data 2. DNA fragments were amplified using HotStarTaq DNA Polymerase (Qiagen, Redwood City, CA, USA), purified using ExoSAP-IT PCR Purification Kit (USB Corporation, Cleveland, OH, USA), and sequenced using the Big Dye Terminator v3.1 kit (Applied Biosystems, Foster City, CA, USA). Sanger sequencing was performed on an ABI3130 Sequencer (Applied Biosystems), and visualised in Geneious 5.6.2 software (BioMatters Ltd., Auckland, New Zealand).

### Tumour micro-dissection and analysis

Both tumours were reviewed by a clinical pathologist with expertise in this area. Consecutive 10 μm sections were cut from the formalin fixed paraffin embedded PMP specimens with the highest tumour content, and stained with haematoxylin and eosin. Tumour cells were micro-dissected manually using a 23 gauge needle and somatic DNA was extracted using the DNeasy Blood and Tissue Kit (Qiagen, Redwood City, CA, USA). Somatic copy number analysis of tumours was assayed using the OncoScan Molecular Inversion Probe assay (*Affymetrix,* Santa Clara, CA, USA) on 50–75 ng of somatic DNA, and the data analysed using Nexus Copy Number™ software (Biodiscovery, Inc., El Segundo, CA, USA). There was no matched control copy number data available for P2. The Oncoscan molecular assay comprises > 220,000 single nucleotide polymorphisms and provides copy number resolution of around 50–100 kb.

To assess if a variant showed somatic LOH, Sanger sequencing was performed using unamplified tumour DNA extracted from the AMT (primers listed in Supplementary Data 3).

### Sanger sequencing of candidate genes in an AMT/PMP validation cohort

Germline DNA from individuals with AMTs or PMP was obtained from the Victorian Cancer Biobank (VCB), the Australian Ovarian Cancer Study (AOCS) and Southampton, UK [31]. Clinical details were extracted from de-identified histopathology reports. Histopathology reports for all PMP samples in the validation cohort were examined to ensure that they were not metastases of known ovarian origin.

Sanger sequencing of all exons of *REEP5* was performed using germline DNA from individuals from the PMP validation cohort, using the same methods as described earlier. PCR primers for each exon were designed to include 40 base pairs flanking the intron-exon boundary of each exon (Supplementary Data 4).

## Results

### Analysis of genes associated with known familial colorectal cancer syndromes

Prior to analysis for novel shared germline variants, a targeted analysis was undertaken of 17 genes associated with an increased familial colorectal cancer risk*: APC, MUTYH, MLH1, MSH2, MSH6, PMS2, BMPR1A, SMAD4, STK11, EPCAM* [32]*, GREM1, POLE* [33]*, POLD1* [33]*, BUB1* [34]*, BUB1B* [35]*, BUB3* [34] *and NTHL1* [36]*.* All these genes excluding *STK11* were sequenced with a mean coverage of >68X (Supplementary Data 5). No LoF or known pathogenic missense variants were identified in either case.

### Identification of shared germline variants

LoF and missense variants with an allele frequency between 0.15 and 0.8 were selected in order to identify heterozygous variants. A total of 4893 and 4973 variants were identified in P1 and P2, respectively. After excluding any variant previously reported in the 1000 Genomes Project or the NHLBI GO Exome Sequencing Project, or a local cohort of 147 familial breast cancer exomes, a total of 106 and 110 variants remained in P1 and P2, respectively. Of these, 40 variants (8 LoF and 32 missense) were shared between P1 and P2. Manual curation of the variant reads in IGV eliminated a further 9 variants as likely artefacts due to misalignment, known hypervariable genes or variants located in areas with low mapping quality. Missense variants were prioritised on their CADD score. A CADD scaled score of ≥10 was used as an inclusion threshold, as this identifies the top 10% most deleterious substitutions in the human genome [25]. This approach eliminated a further seven missense variants. Following validation by Sanger sequencing and verification of allele frequencies in the gnomAD dataset, the final list of shared variants in 15 genes comprised 1 LoF and 14 missense variants (Table 1). The number of variants remaining after each filtering step is summarised in Supplementary Data 6.

**Table 1:** Fifteen validated variants seen in both P1 and P2

Gene	Ref_Seq mRNA transcript	cDNA variant	Amino Acid Change	Variant type	Scaled CADD score	variant frequency in gnomAD v2
***REEP5***	NM_005669.4	c.159 T > G	p.Tyr53*	Nonsense	31	0
***RHBDL2***	NM_017821.3	c.395C > G	p.Gly132Ala	Missense	29.8	0
***FGFR4***	NM_022963.2	c.1988G > A	p.Arg663Gln	Missense	21.9	4.42E-05
***CNTN2***	NM_005076.3	c.2263A > T	p.Ser755Cys	Missense	21.2	3.98E-06
***RANBP2***	NM_006267.4	c.3683G > T	p.Gly1228Val	Missense	19.8	0
***ZNF747***	NM_023931.2	c.254C > T	p.Gly85Glu	Missense	16.28	5.70E-06
***EXOG***	NM_005107.3	c.178G > A	p.Ala60Thr	Missense	16.17	0
***BRINP3***	NM_199051.1	c.809A > C	p.Glu270Ala	Missense	16.1	1.60E-05
***ASIC1***	NM_020039.3	c.463C > T	p.Arg155Cys	Missense	16.05	0
***TNFRSF1B***	NM_001066.2	c.1337A > G	p.Glu446Gly	Missense	15.46	3.99E-06
***RANBP6***	NM_001243202.1	c.251A > C	p.Glu84Ala	Missense	14.87	3.98-E06
***LSR***	NM_205834.3	c.725C > T	p.Thr242Ile	Missense	14.79	3.20E-05
***MTERFD3***	NM_001033050.1	c.607C > T	p.Ala203Thr	Missense	12.04	1.20E-05
***PAX1***	NM_001257096.1	c.1379G > T	p.Arg460Leu	Missense	11.57	5.11E-05
***EGFR***	NM_005228.3	c.1915A > C	p.ASN639His	Missense	10.51	0

*CADD* Combined Annotation Dependent Depletion Score, *gnomAD v2* Genome Aggregation Database version 2

### Identification of germline variants associated with concomitant areas of tumour LOH

Assuming a possible two-hit model for germline tumour suppressor inactivation, analysis was performed to identify germline candidate variants present in regions of LOH in the tumours.

**Fig. 2:**
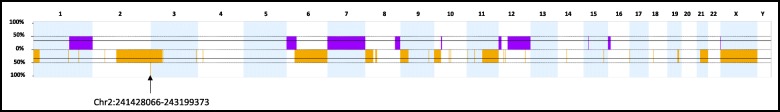
Summary plot of combined regions of copy number gain (blue) or loss of heterozygosity (brown). Legend. Copy number gain (blue) or loss of heterozygosity (brown), with bars reaching 100% indicating copy number aberrations seen in both tumours. The region on chromosome 2 containing loss of heterozygosity in both tumours is indicated by the arrow, with genomic coordinates listed below arrow. Chr, chromosome

The Oncoscan array detected somatic LOH (copy number neutral and loss) in 20.3% and 19.8% of the tumour genomes of P1 and P2, respectively (Tables 2 and 3). Only one region of LOH, a 1.77 Mb region on chromosome 2q, was common to both tumours (Fig. 2). This region contains 33 genes but none harboured likely pathogenic germline variants common to both individuals.

**Table 2:** Regions of loss of heterozygosity (LOH) and copy number (CN) loss in pseudomyxoma peritonei tumour from P1

Chromosome	Start	End	Event
1	0	26,948,921	CN Loss/LOH
1	147,134,028	147,825,662	LOH
1	190,881,536	191,887,248	LOH
2	50,914,938	51,446,707	LOH
2	99,377,108	243,199,373	LOH
3	0	48,040,095	LOH
3	50,384,337	52,768,237	LOH
3	191,022,128	191,856,841	LOH
4	21,562,094	22,526,745	LOH
6	33,506,076	171,115,067	CN Loss/LOH
7	22,818,202	23,530,679	LOH
9	0	33,946,637	LOH
9	118,867,206	119,530,524	LOH
10	62,401,757	63,554,609	LOH
10	69,912,047	70,990,019	LOH
11	5,529,179	6,091,608	LOH
11	34,356,991	35,043,999	LOH
12	21,011,988	22,928,787	LOH
12	29,116,987	29,816,788	LOH
12	111,166,777	112,632,998	LOH
14	36,158,966	36,786,121	LOH
15	84,783,628	85,501,061	LOH
17	857,820	1,452,131	LOH
18	18,535,946	19,513,726	LOH
19	41,994,722	48,382,347	CN Loss/LOH
21	14,414,872	48,129,895	CN Loss/LOH

*CN* copy number, *LOH* loss of heterozygosity

**Table 3:** Regions of loss of heterozygosity (LOH) and copy number (CN) loss in pseudomyxoma peritonei tumour from P2

Chromosome	Start	End	Event
2	241,428,066	243,199,373	CN Loss/LOH
8	0	33,395,041	CN Loss/LOH
8	42,137,047	49,106,261	LOH
10	0	29,922,641	CN Loss
11	66,296,148	135,006,516	LOH
11	111,333,148	117,815,955	CN Loss
11	130,316,743	135,006,516	CN Loss
X	1	155,270,560	CN Loss/LOH

CN, copy number. LOH, loss of heterozygosity

As LOH is not the only mechanism by which a somatic wild type allele can be abrogated, the analysis was extended to incorporate all regions of somatic LOH present in either P1's or P2’s tumour. Missense variants in four genes, *EXOG*, *RANBP2, RANBP6 and TNFRSF1B*, were identified in areas of LOH seen in the tumour from P1. The LOH regions in the tumour from P2 did not harbour any shared germline variants.

The variants in *EXOG*, *RANBP2, RANBP6 and TNFRSF1B* were sequenced in the tumour DNA of P1 to assess which allele of the gene was lost. *RANBP2* showed somatic loss of the mutant allele. Unexpectedly, somatic sequencing of *EXOG*, *RANBP6* and *TNFRSF1B* demonstrated the presence of both the wild type and mutant alleles (Supplementary Data 7 and 8). These discordant results may relate to the genomic heterogeneity within subclones of the PMP tumours. The DNA used as template for Sanger sequencing was extracted from the primary appendiceal tumour whilst the template DNA for the Oncoscan array was extracted from secondary peritoneal tumour in order to obtain sufficient DNA. Due to the sparse nature of the tumour cells, no remaining DNA was available for Sanger sequencing from the secondary peritoneal tumour.

### Screening of the *REEP5* gene in a validation cohort of individuals with presumed sporadic AMTs/PMP using Sanger sequencing

Having identified a LOF variant in *REEP5* present in both P1 and P2, further independent evidence implicating this gene in AMT predisposition was found in a previous case report [37] of an individual with a chromosomal translocation t(5;8)(q22;p23.1) who was diagnosed with a mucin-secreting appendiceal carcinoma and familial adenomatous polyposis (FAP) at the age of 26 years. Fluorescent in situ hybridisation studies in this case identified a microdeletion encompassing both APC and MCC genes, and by implication the intervening gene *REEP5* (also known as *DP1*). On the basis of this additional information, Sanger sequencing of the five exons of *REEP5* (NM_005669.4) was performed on the germline DNA of 23 individuals with presumed sporadic AMTs (n = 13) and/or PMP of presumed appendiceal origin (Table 4). The age of diagnosis (extracted from the histopathology report) ranged from 31 to 72 years, with a median age of 58 years. The age for one individual was unknown. No LoF variants or missense variants (excluding common polymorphisms) in *REEP5* were identified.

**Table 4:** Characteristics of the validation cohort of AMTs and/or PMPs

Characteristic	Number
Validation Cases	23
Male	12
Female	11
AMTs	13
LAMN	7
Appendiceal mucinous adenocarcinomas	5
HAMN	1
Associated with PMP	10
PMP only	10
Low grade	8
High grade	1
Mixed	1
Implied history of AMT^#^	4

*AMTs* appendiceal mucinous tumours, *LAMN* low-grade appendiceal mucinous neoplasm, *HAMN* high grade appendiceal mucinous neoplasm, *PMP* pseudomyxoma peritonei. #history of AMT implied in histopathology report

## Discussion

As PMP is a rare disorder, we postulated that this apparent familial occurrence of the disease in a father and daughter might imply a hereditary predisposition*.* Only a limited assessment of the known colorectal cancer predisposing genes had been performed previously on familial PMP cases in the literature. Through germline whole exome sequencing of both affected individuals in our parent-child PMP family, we were able to assess and exclude the known colorectal cancer predisposing genes including *APC, MLH1, MSH2, MSH6, PMS2, MUTYH, BMPR1A, SMAD4, POLE and POLD1*, with the caveat that *STK11* could not be excluded due to low coverage on exome sequencing.

We took an agnostic approach to identifying the causative gene by identifying novel (ie. not present in the 1000 Genomes Project and NHLBI GO Exome Sequencing Project datasets) shared germline variants in the exome of both individuals, which were also very rare (≤1X10^–4^ in the gnomAD dataset), coupled with genome-wide copy number analysis of both tumours. We identified 15 potentially pathogenic variants shared between the two cases, including four within regions of LOH in the father’s tumour. Loss of the potentially pathogenic missense variant in *RANBP2* in the tumour genome, suggests this variant is unlikely to be the cause of the PMP predisposition. The missense variants in *EXOG*, *RANBP6* and *TNFRSF1B* remained heterozygous in the primary tumour of P1, although with the loss of the second allele at a later stage, this does not necessarily exclude these variants as possible PMP predisposition genes on these data alone.

A shared nonsense variant in *REEP5* p.Tyr53* *(*NM_005669.4:c.159 T > G) is a plausible candidate predisposing variant, although both tumours retained heterozygosity at this locus. *REEP5* has been implicated in the regulation of *TP53*, a known cancer predisposition gene, through its interaction with *HCCR1* [38, 39]. It is expressed in normal colonic tissue and has been shown to be down-regulated in colon cancers. Similarly, transfection of *REEP5* into RKO colon cancer cells results in growth inhibition and induction of apoptosis, suggesting it functions as a tumour suppressor [38]. *REEP5* is also involved in stabilisation of the endoplasmic reticulum tubules via its effect on the curvature of the endoplasmic reticulum lipid bilayer [40]. The lack of *REEP5* mutations in the 23 validation cases that were screened suggests that germline *REEP5* mutations are not common in sporadic AMTs/PMPs, however this is a small dataset of a very rare tumour type, and more cases are required to determine if *REEP5* is implicated in the development of AMTs.

Several genes that contained a shared rare missense variant have functions consistent with cancer predisposition. *RHBDL2* is a rhomboid intra-membrane protease that activates epidermal growth factor and is associated with anoikis resistance [41]. *FGFR4* is part of the fibroblast growth factor receptor family, a subset of tyrosine kinase receptors that are highly conserved. The fibroblast growth factor receptors have been extensively investigated somatically in relation to various cancers, and several fibroblast growth factor receptor inhibitors are in clinical trials [42]. The functional consequence of this variant is unknown, however it has a high CADD scaled score and is predicted to be deleterious by SIFT and potentially damaging by PolyPhen, making it an interesting candidate for further functional study. A common germline polymorphism (*FGFR4:*c.1162G > A) has been associated with an increased risk of developing breast and prostate cancers [43]. *EGFR* is a receptor tyrosine kinase that is somatically mutated in 10% of non-small cell lung cancers [44]. Exon 19 deletions and a p.L858R mutation account for approximately 90% of such mutations, and predict exquisite sensitivity to EGFR tyrosine kinase inhibitors [45–47]. The identified missense variant in this study lies outside the protein kinase domain, hence a potential role in the development of AMTs remains to be determined.

There are a number of limitations with this study. This study only searched exon-based sequences and as such would be unable to identify sequence variants present in gene regulatory regions. The aggregation of PMP within the family may also have arisen due to a shared environmental factor, or due to stochastic events. In view of these limitations, it is possible that the shared variants identified in this study may reflect shared private variants unrelated to a predisposition to AMTs.

The validation cohort consisted of 3 cases ascertained on AMTs without associated PMP on the histopathology report. Conversely for the cases ascertained on PMP alone, an appendiceal primary was not present in the histopathology report, but was alluded to in 4 cases and assumed for the remaining cases. As it is unknown if all AMTs give rise to PMP, further studies of *REEP5* in AMTs with known associated PMP may be required to determine if *REEP5* mutations predispose to AMTs associated with PMP.

With regards to the use of LOH to identify second hits in candidate genes, PMP is a hypo-cellular tumour with only widely scattered groups of tumour cells in abundant mucin. As there was insufficient DNA in the primary tumours for genome wide copy number analysis, DNA was obtained from associated PMPs. This phenotype provided technical problems in the somatic LOH analysis.

## Conclusions

Through germline whole exome sequencing of a very rare familial occurrence of a very rare cancer type, in which only 2 families with limited genomic analyses have been reported in the literature, our study makes several contributions to knowledge.

We have identified candidate variants that may predispose to the development of AMTs and PMP in this family. We were also able to examine the known CRC-predisposing genes (apart from *STK11)* and found no known pathogenic variants in this family, suggesting that other novel genes may predispose to this rare subtype of cancer in the colon. Through the re-sequencing of *REEP5* in 23 sporadic AMTs and/or PMP cases, we did not identify any germline *REEP5* mutations, despite a shared LoF variant found in our family, and the loss of *REEP5* in a case report of a patient with FAP and an appendiceal mucinous tumour. Further studies are required to ascertain if the genes identified in this study play a role in the development of PMP in familial or sporadic cases.

## Supplementary information

**Additional file 1: Supplementary Data 1.** Ranking of gene transcript according to predicted functional consequence of a variant.pdf. **Supplementary Data 2.** PCR primers for validation of loss-of-function (LoF) and missense variants with Combined Annotation Dependent Depletion scaled score ≥ 10.pdf. **Supplementary Data 3.** M13 PCR primers for determination of loss-of-heterozygosity (LoH) in tumour tissue.pdf. **Supplementary Data 4.** Primers for *REEP5* exons.pdf. **Supplementary Data 5.** Coverage of familial colorectal cancer genes in whole exome sequencing.pdf. **Supplementary Data 6.** Number of variants at each step of the filtering process.pdf. **Supplementary Data 7.** Sanger sequencing traces for the *RANBP2* missense variant in the germline of P1 (top left), germline of P2 (middle left), and tumour of P1 (bottom left), showing loss of the mutant allele.pdf. **Supplementary Data 8.** Sanger sequencing traces for missense variants in *EXOG* (top panel)*, RANBP6* (middle panel) and *TNFRSF1B* (bottom panel) showing the presence of the variant in the germline of P1, P2 and in tumour from P1, despite the presence of loss of heterozygosity of approximately a)50Mbs b)30Mbs and c) loss of heterozygosity and copy number loss of approximately 20Mbs.pdf.

## Data Availability

The datasets used and/or analysed during the current study are available from the corresponding author on reasonable request.

## Abbreviations

AMT: Appendiceal mucinous tumour
CADD: Combined Annotation Dependent Depletion
DNA: Deoxyribonucleic acid
FAP: Familial adenomatous polyposis
GATK: Genome Analysis Tool Kit
gnomAD: Genome Aggregation Database
HAMN: High grade appendiceal mucinous neoplasm
LAMN: Low-grade appendiceal mucinous neoplasm
LoF: Loss-of-function
LOH: Loss of heterozygosity
NHLBI GO: National Heart, Lung and Blood Institute Grand Opportunity
PCR: Polymerase chain reaction
PMP: Pseudomyxoma peritonei
SNV: Single nucleotide variant
