# Supplementary material for: Germline whole exome sequencing of a family with appendiceal mucinous tumours presenting with pseudomyxoma peritonei
Source: BMC Cancer. 2020 May 1;20:369. doi: 10.1186/s12885-020-6705-y (PMC7195761; doi:10.1186/s12885-020-6705-y)
Supplement: Supplementary file 1 — Additional file 1: Supplementary Data 1. Ranking of gene transcript according to predicted functional consequence of a variant.pdf. Supplementary Data 2. PCR primers for validation of loss-of-function (LoF) and missense variants with Combined Annotation Dependent Depletion scaled score ≥ 10.pdf. Supplementary Data 3. M13 PCR primers for determination of loss-of-heterozygosity (LoH) in tumour tissue.pdf. Supplementary Data 4. Primers for REEP5 exons.pdf. Supplementary Data 5. Coverage of familial colorectal cancer genes in whole exome sequencing.pdf. Supplementary Data 6. Number of variants at each step of the filtering process.pdf. Supplementary Data 7. Sanger sequencing traces for the RANBP2 missense variant in the germline of P1 (top left), germline of P2 (middle left), and tumour of P1 (bottom left), showing loss of the mutant allele.pdf. Supplementary Data 8. Sanger sequencing traces for missense variants in EXOG (top panel), RANBP6 (middle panel) and TNFRSF1B (bottom panel) showing the presence of the variant in the germline of P1, P2 and in tumour from P1, despite the presence of loss of heterozygosity of approximately a)50Mbs b)30Mbs and c) loss of heterozygosity and copy number loss of approximately 20Mbs.pdf. [file 12885_2020_6705_MOESM1_ESM.docx]

Supplementary Data

**Germline whole exome sequencing of a family with appendiceal mucinous tumours presenting with pseudomyxoma peritonei**

Mei Sim Lung^1^, Catherine A. Mitchell^2^, Maria A. Doyle^3^, Andrew C. Lynch^4^, Kylie L. Gorringe^5,7^, David D.L. Bowtell ^6,7,8^, Australian Ovarian Cancer Study Group, Ian G. Campbell^1,2,7¶^, Alison H. Trainer^1,9,10¶^

^1^Cancer Genetics Laboratory, Peter MacCallum Cancer Centre, Melbourne , Victoria, Australia

^2^Department of Pathology, Peter MacCallum Cancer Centre, Melbourne, Victoria, Australia

^3^Research Computing Facility, Peter MacCallum Cancer Centre, Melbourne, Victoria, Australia

^4^Department of Surgical Oncology, Peter MacCallum Cancer Centre, Melbourne, Victoria, Australia

^5^ Cancer Genomics Program, Peter MacCallum Cancer Centre, Melbourne, Victoria, Australia

^6^Cancer Genetics and Genomics Laboratory, Peter MacCallum Cancer Centre, Melbourne, Victoria, Australia

^7^Sir Peter MacCallum Department of Oncology, University of Melbourne, Parkville, Victoria, Australia

^8^Department of Biochemistry and Molecular Biology, University of Melbourne, Parkville, Victoria, Australia

^9^ Parkville Familial Cancer Centre, Peter MacCallum Cancer Centre, Melbourne, Victoria, Australia

^10^ Department of Medicine, University of Melbourne, Parkville, Victoria, Australia

^¶^ co-senior authors

**Corresponding author**

Ian G. Campbell

Cancer Genetics Laboratory

Research Division

Peter MacCallum Cancer Centre

Victorian Comprehensive Cancer Centre Building

305 Grattan St

Melbourne VIC 3000

Australia

Tel no:(+613) 85597109

E-mail address:ian.campbell@petermac.org

ORCID ID: 0000-0002-7773-4155

**Supplementary Data 1. Ranking of gene transcript according to predicted functional consequence of a variant**

| **Functional Consequence** | **Rank** |
| --- | --- |
| Nonsense, frameshift | 1 |
| Essential Splice Site | 2 |
| Missense | 3 |
| Other | 4 |

**Supplementary Data 2. PCR primers for validation of loss-of-function (LoF) and missense variants with Combined Annotation Dependent Depletion scaled score ≥10**

| **Gene** | **Sequencing direction** | **Primer Sequence** | **PCR Product Size** |
| --- | --- | --- | --- |
| *RHBDL2* | Forward | CTGAGAAGAGGGAGGAAGCC | 244 |
| *RHBDL2* | Reverse | cctcagcctccatgactaca | 244 |
| *FGFR4* | Forward | TGGTCTTTTGGGATCCTGCT | 185 |
| *FGFR4* | Reverse | ggaggaggaggactggaaag | 185 |
| *CNTN2* | Forward | cttggcaaaggttgggtgg | 203 |
| *CNTN2* | Reverse | GACAAAGTACTGGGCATCGG | 203 |
| *RANBP2* | Forward | AAGAATGGAAAGAACGTGGGA | 220 |
| *RANBP2* | Reverse | GCAAGTTGTTCTGGTTTTGGC | 220 |
| *ZNF747* | Forward | GAGACCTACGGCCACCTG | 225 |
| *ZNF747* | Reverse | ACAGTTCGGCCTTCTCCTC | 225 |
| *BRINP3* | Forward | tgttcttcaagGGCTTCAAGT | 150 |
| *BRINP3* | Reverse | TCTGGAAATTTGGGACCACAG | 150 |
| *ASIC1* | Forward | GATGGCAGATGAAAAGCAGC | 244 |
| *ASIC1* | Reverse | gcctgctccttgtctcctc | 244 |
| *TNFRSF1B* | Forward | CTTTCGGTCACAGCTGGAGA | 226 |
| *TNFRSF1B* | Reverse | GAACTTGGCCCAGAAAGAGC | 226 |
| *RANBP6* | Forward | ACCTTCCTCTTAGATGCCGT | 236 |
| *RANBP6* | Reverse | ATCAAATTCCTGGCCAGCAC | 236 |
| *LSR* | Forward | ccatgcactagggcttcag | 237 |
| *LSR* | Reverse | ctcccgagaagcccaactc | 237 |
| *MTERFD3* | Forward | TGACAGCTGCACCTAATGTT | 206 |
| *MTERFD3* | Reverse | ACCTTGCTCCTGGAGAAATTC | 206 |
| *PAX1* | Forward | gtagtgatccgacgcctctg | 239 |
| *PAX1* | Reverse | CTCCGCGAGTCCTCCGATC | 239 |
| *EGFR* | Forward | tctctttcacttcctacagATGC | 150 |
| *EGFR* | Reverse | aggacagtcagaaatgcagga | 150 |

**Supplementary Data 3. M13 PCR primers for determination of loss-of-heterozygosity (LoH) in tumour tissue**

| **Gene** | **Sequencing direction** | **Primer Sequence** | **PCR Product Size** |
| --- | --- | --- | --- |
| *REEP5** | Forward | GTAAAACGACGGCCAGTttgtggtccccagGTGTC | 108 |
| *REEP5** | Reverse | AACAGCTATGACCATGcGAGATGTAGGCTGGGTAGC | 108 |
| *EXOG** | Forward | GTAAAACGACGGCCAGTaagagccactaattgtgtgtgtg | 136 |
| *EXOG** | Reverse | AACAGCTATGACCATGACACCTTGCCTCTGTTCCAG | 136 |
| *RANBP2* | Forward | GTAAAACGACGGCCAGTATGGAAAGAACGTGGGATTG | 108 |
| *RANBP2* | Reverse | AACAGCTATGACCATGTTGCACAGATTTTCAATACTTGCT | 108 |
| *RANBP6* | Forward | GTAAAACGACGGCCAGTACAAATGGCTGCCGCACT | 113 |
| *RANBP6* | Reverse | AACAGCTATGACCATGCAGCCAGAATCAGTTCAATCTT | 113 |
| *TNFRSF1B* | Forward | GTAAAACGACGGCCAGTAATGTGCCTTTCGGTCACAG | 110 |
| *TNFRSF1B* | Reverse | AACAGCTATGACCATGTGGTTAACTGGGCTTCATCC | 110 |
| M13 Forward Primer | | GTAAAACGACGGCCAGT | N/A |
| M13 Reverse Primer | | AACAGCTATGACCATG | N/A |

*Primers also used for validation of variants in germline DNA

**Supplementary Data 4. Primers for *REEP5* exons**

| **Exon** | **Sequencing direction** | **Primer Sequence** | **PCR Product Size** |
| --- | --- | --- | --- |
| Exon 1 | Forward | AGTCGCCGCTCCAGTCTAT | 266 |
| Exon 1 | Reverse | gagtcctctccctgcttcct | 266 |
| Exon 2 | Forward | cgagtcgagaggaaaactcc | 292 |
| Exon 2 | Reverse | aattgcggatgtgcagtcta | 292 |
| Exon 3 | Forward | gaaagtgtatttgaaaatctttcattg | 298 |
| Exon 3 | Reverse | gccacagatgcacaacattc | 298 |
| Exon 4 | Forward | cccatccccagagtagtgaa | 364 |
| Exon 4 | Reverse | gctgaggggtggtgataaga | 364 |
| Exon 5 | Forward | atgatcgcacctgaacatga | 287 |
| Exon 5 | Reverse | TGTTTCCAAGGCAACATTATT | 287 |

**Supplementary Data 5. Coverage of familial colorectal cancer genes in whole exome sequencing**

| **Sample** | **Gene** | **Mean coverage** |
| --- | --- | --- |
| **P1** | *APC* | 74.59 |
| **P2** | *APC* | 73.25 |
| **P1** | *BMPR1A* | 154.82 |
| **P2** | *BMPR1A* | 201.13 |
| **P1** | *EPCAM* | 80.65 |
| **P2** | *EPCAM* | 93.60 |
| **P1** | *GREM1* | 68.52 |
| **P2** | *GREM1* | 92.03 |
| **P1** | *MLH1* | 158.03 |
| **P2** | *MLH1* | 138.76 |
| **P1** | *MSH2* | 108.17 |
| **P2** | *MSH2* | 105.14 |
| **P1** | *MSH6* | 76.81 |
| **P2** | *MSH6* | 75.05 |
| **P1** | *MUTYH* | 125.69 |
| **P2** | *MUTYH* | 88.85 |
| **P1** | *NTHL1* | 172.70 |
| **P2** | *NTHL1* | 127.78 |
| **P1** | *PMS2* | 129.68 |
| **P2** | *PMS2* | 130.68 |
| **P1** | *POLD1* | 96.10 |
| **P2** | *POLD1* | 85.39 |
| **P1** | *POLE* | 193.59 |
| **P2** | *POLE* | 186.60 |
| **P1** | *SMAD4* | 205.47 |
| **P2** | *SMAD4* | 240.61 |
| **P1** | *STK11* | 3.46 |
| **P2** | *STK11* | 2.08 |

**Supplementary Data 6. Number of variants at each step of the filtering process**

| **Sample**  **ID** | **Non synonymous variants with an allele frequency 0.15 - 0.80** | **Variants not seen in 1000G or NHLBI GO ESP** | **Variants post local exomes filter(n=147)** | **Variants shared by both cases** | **Shared variants excluding missense variants with a CADD score<10** | **Sanger sequenced validated variants with a gnomAD allele frequency of ≤1X10^-4^** |
| --- | --- | --- | --- | --- | --- | --- |
| **P1** | 4893 | 586 | 106 | 40 | 24 | 15 |
| **P2** | 4973 | 678 | 110 |  |  |  |

1000G,1000 Genomes Project. NHLBI GO ESP, National Heart, Lung and Blood Institute Exome Sequencing Project.


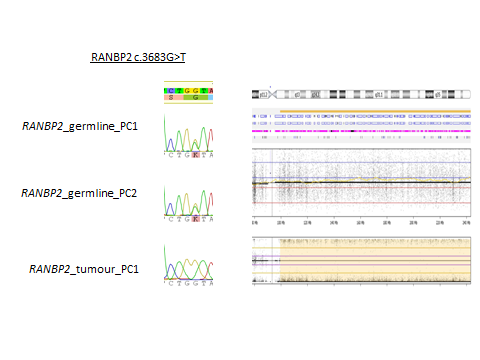


**P1**1[Type a quote from the document or the summary of an interesting point. You can position the text box anywhere in the document. Use the Drawing Tools tab to change the formatting of the pull quote text box.]

**P2**

**P1**

**Supplementary Data 7. Sanger sequencing traces for the *RANBP2* missense variant in the germline of P1 (top left), germline of P2 (middle left), and tumour of P1 (bottom left), showing loss of the mutant allele.**


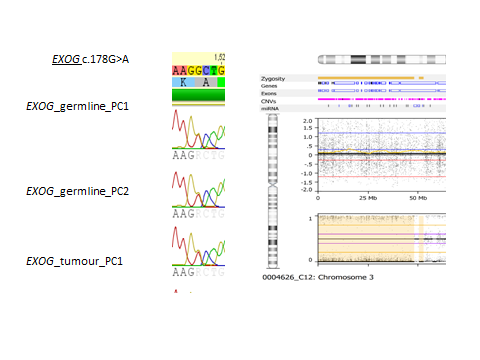

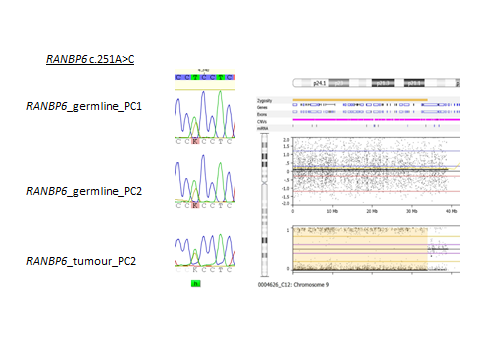

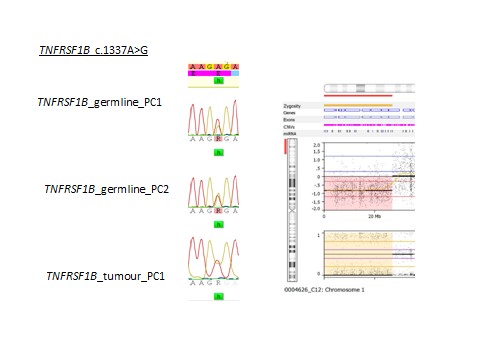


**P2**

**P1**

**P1**

**P1**

**P2**

**P1**

**P2**

**P1**

**P1**

**Supplementary Data 8. Sanger sequencing traces for missense variants in *EXOG* (top panel)*,* *RANBP6* (middle panel) and *TNFRSF1B* (bottom panel) showing the presence of the variant in the germline of P1, P2 and in tumour from P1, despite the presence of loss of heterozygosity of approximately a)50Mbs b)30Mbs and c)loss of heterozygosity and copy number loss of approximately 20Mbs.**
